# Safety and feasibility of a rapid reversible induction strategy in anesthesia induction

**DOI:** 10.1016/j.jatmed.2025.03.002

**Published:** 2025-05-19

**Authors:** Xiaoying Chi, Yichen Fan, Xiao Zhang, Yi Qin, Jie Xiao, Zhenling Huang, Diansan Su

**Affiliations:** a Department of Anesthesiology, Renji Hospital Shanghai Jiaotong University School of Medicine, Shanghai 200127, China; b Department of Anesthesiology, The First Affiliated Hospital, Zhejiang University School of Medicine, Hangzhou 310003, China

**Keywords:** Difficult airway, Guidelines for difficult airway, High-flow nasal oxygenation, Rapid reversible induction, The rate of reversion success

## Abstract

**Background:**

Unexpected airway management challenges during anesthesia induction—particularly "Cannot Intubate, Cannot Ventilate" (CICV) scenarios—pose significant risks of hypoxemia and hemodynamic instability. Rapid reversal of anesthesia to restore adequate spontaneous ventilation represents a critical clinical objective. Our research focuses on developing safe and rapidly reversible induction strategies, which aim to provide alternative solutions for difficult airway management before the onset of irreversible intubation or ventilation failure.

**Methods:**

High-flow oxygen inhalation (40 L·min^−1^) and FiO_2_ (100 %) were administered preoperatively for five minutes. During anesthesia induction, target controlled infusion was performed with remifentanil 4 ng·mL^−1^ and propofol 4 μg·mL^−1^, and rocuronium 0.6 mg·kg^−1^ was injected intravenously. Subsequently, the oxygen flow was adjusted to 60 L·min^−1^, with the patients’ jaws lifted after the patients had lost consciousness. A direct laryngoscope was used for laryngoscopy and grading. The tracheas of patients with Cormack–Lehane (CL) grade I–II were intubated directly. For the patients with CL grade III–IV, sugammadex (16 mg·kg^−1^) was administered and TCI was stopped. Then conscious intubation was performed after the patients were fully awake. The primary outcome was the rate of reversion success within 15 min from induction.

**Results:**

In total, 20 patients with CL grade 3–4 undergoing elective surgery needed reversion. The reversion success rate was 100 %. The shortest time to reversion was 333 s, the longest was 900 s, and the median time was 455 s. During the whole procedure, no hypoxia occurred, and acceptable hypercapnia appeared.

**Conclusions:**

The findings of this study demonstrate that the rapid reversible induction (RRI) strategy is both safe and technically feasible. Prompt reversal of anesthesia to restore spontaneous ventilation should be prioritized as a proactive intervention in cases of unanticipated difficult airways, before the situation progresses to a "Cannot Intubate, Cannot Ventilate" (CICV) crisis.

## Introduction

Airway management in anesthesia carries inherent risks, with the "Cannot Intubate, Cannot Ventilate" (CICV) scenario posing a critical threat to patient safety. CICV often arises gradually, and proactive reversal of anesthesia before its onset is a rational strategy to mitigate hypoxemia risks. Maintaining oxygenation during this reversal process is essential to extend the safe apnea window and allow time for alternative interventions.^1^, ^2^

To address this challenge, we propose a rapidly reversible induction strategy (RRI) that integrates pharmacologically optimized agents and advanced preoxygenation techniques. The selection of short-acting anesthetics—propofol, remifentanil, and rocuronium—alongside sugammadex for the rapid neuromuscular blockade reversal, forms the cornerstone of this strategy. Propofol provides rapid induction with minimal airway reflex activation, while remifentanil attenuates sympathetic responses without prolonging recovery.^2^, ^3^ Rocuronium, a non-depolarizing neuromuscular blocker, offers a predictable onset and is fully reversible by sugammadex within minutes, even in deep blockade.^4^ This combination ensures both rapid induction and emergency reversibility, which are critical for avoiding CICV escalation.

Preoxygenation is pivotal to prolong the safe apnea duration. High-Flow Nasal Oxygenation delivers heated, humidified oxygen at flows up to 60 L·min^−1^, generating positive airway pressure (4–6 cmH_2_O) that enhances functional residual capacity and reduces atelectasis. Studies demonstrate that HFNO extends the duration of apnea without desaturation by 125 s compared to conventional methods, particularly in obese or critically ill patients. Moreover, its non-invasive design minimizes interference during intubation attempts, making it ideal for high-risk populations.^5^, ^6^ The integration of HFNO with rapid-acting, reversible agents addresses the dual imperatives of oxygenation and flexibility. RRI exemplifies a paradigm shift toward safer, adaptable anesthesia practices.

## Materials and methods

This study was approved by the Renji Hospital Ethics Committee (RenJiH[2018]01) and registered at ClinicalTrials.gov (NCT04434963). This prospective clinical case series study was conducted at Renji Hospital, Shanghai Jiao Tong University School of Medicine. All investigators underwent sample training for the high-flow nasal oxygen (HFNO) setup (Fisher & Paykel, Panmure, New Zealand).

### Study population

From February 2021 to December 2021, 20 patients with Cormack–Lehane (CL) grade 3–4 were enrolled among 121 patients with Mallampati classification III-IV.

Patients undergoing elective surgery (estimated operation time > 2 h) were enrolled in this study. All patients provided written informed consent. The inclusion criteria were (1) patients aged >18 years and <65 years; (2) provision of signed informed consent; (3) patients undergoing elective operation (operation time >2 h); (4) American Society of Anesthesiologists (ASA) classification I–II; (5) Mallampati classification III-IV; 6) suspected difficult airway for which an anesthesiologist performed rapid anesthesia induction. The exclusion criteria were (1) liver, kidney, or heart dysfunction; (2) presence of a full stomach or other risk factors of reflux aspiration; (3) use of medication that may affect neuromuscular blockade, (4) presence of mouth or nose infection; (5) history of allergy to any medication used in the study; (6) pregnancy and lactation; and (7) presence of neuromuscular disease.

### Study setting

Electrocardiogram, blood pressure, and oxygen saturation (SpO_2_) were monitored after the patients entered the operating room. A percutaneous radial artery catheter was placed to monitor arterial blood pressure invasively, and arterial blood gas was tested using a blood gas analyzer (ABL90Flex, Radiometer, Denmark). Before anesthesia induction, patients received oxygen (40 L·min^−1^) through HFNO (37 °C, oxygen concentration 100 %) for approximately 5 min. For the induction of anesthesia, target-controlled infusion (TCI, CP660 Beijing Silugao Medical Technology, China) of propofol 4.0 μg·mL^−^^1^ (Marsh model) and remifentanil 4.0 ng·mL^−^^1^ (Minto model) was performed. Rocuronium 0.6 mg·kg^−1^ (Esmeron, Germany) was injected intravenously and oxygen flow was increased to 60 L·min^−1^ (37 °C, oxygen concentration 100 %). No artificial ventilation was performed, but the jaw was raised to keep the airway open after losing consciousness. The depth of anesthesia was detected using an electroencephalogram monitor (Narcotrend, Germany). Neuromuscular blockade was also monitored using a train of four stimulation (TOF, TOF-Watch Sx, Ireland).

Thereafter, a direct laryngoscope was used to determine the CL grade when no simple TOF responses were observed at the adductor pollicis muscle (TOF count, zero) and propofol and remifentanil had reached their target concentrations. Patients with CL grade 1–2 (classified as easy laryngoscopy) were intubated under laryngoscopic guidance, and the patients underwent surgery. Those with CL grade 3–4 (classified as difficult laryngoscopy, as a simulation of CICV conditions) were administered sugammadex (BRIDION, US) 16 mg·kg^−1^, and TCI was stopped. Awake tracheal intubation was performed after the patients were fully reversed. If patients did not experience reversion within 15 min, which is regarded as the absolute safe time of apnea with the high flow according to our previous study,^7^ mask ventilation was performed. After the patients regained consciousness, awake tracheal intubation was performed via fiberoptic bronchoscopy.

**Fig. 1:**
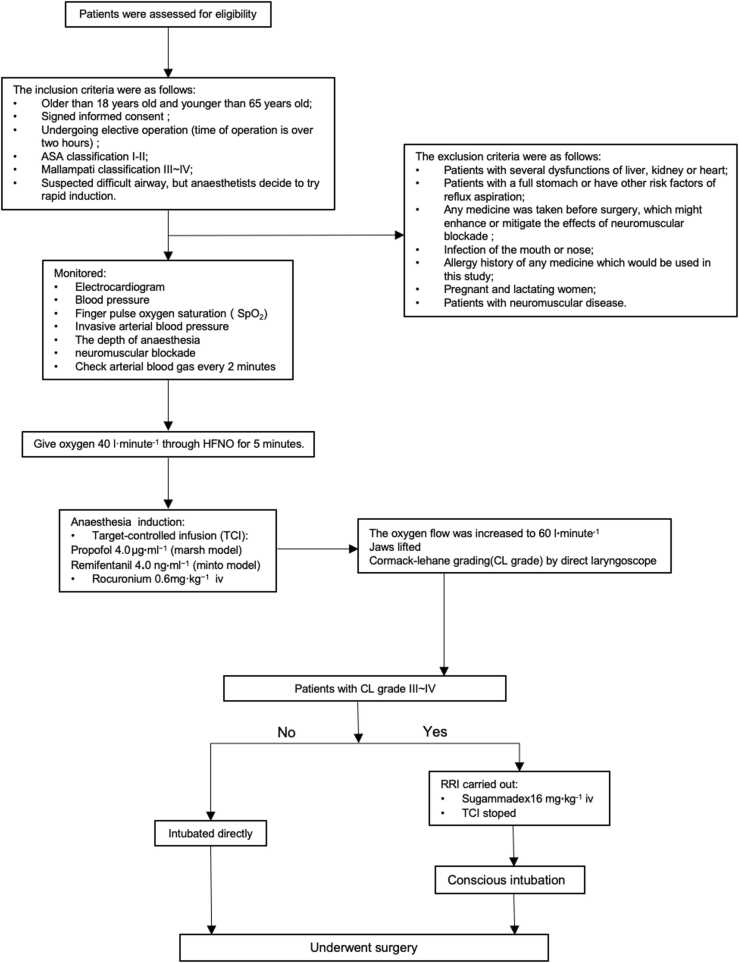
RRI protocol.

Arterial blood gases were assessed every two minutes as a safety index. Heart rate, blood pressure, SpO_2_, TOF ratio, and Narcotrend readings were recorded every five minutes through the entire experimental period as safety indices. The times when the TOF ratio reached zero, TOF ratio > 0.9, and Narcotrend > 80 were recorded. Tidal volume was tested after the recovery of spontaneous breathing using a mask. Other adverse events, including hypoxemia, arrhythmia, and cardiovascular complications, were also recorded. The entire RRI protocol is shown in Fig. 1.

### Primary endpoint

The primary endpoint was the rate of reversion success within 15 min. We recorded the percentage of reversion success within 15 min from the beginning of induction in patients with CL grade 3–4. Successful reversion was defined by spontaneous breathing recovery (tidal volume >6 mL·kg^−1^, calculated according to standard body weight), Narcotrend reading > 80, TOF ratio > 0.9, and patients’ consciousness recovery.

### Secondary endpoints and safety measurements

The secondary endpoints were the time from induction to a TOF ratio over 0.9 and the time from induction to a Narcotrend reading over 80. We measured the time from induction to tidal volume of over 6 mL·kg^−1^ and the time from induction to the recovery of consciousness. The time from the administration of sugammadex to the reversion success was also recorded. Safety measurements included vital signs (e.g., heart rate, blood pressure, SpO_2_, TOF ratio, and Narcotrend readings, and post-anesthesia complications), and hypoxemia, arrhythmia, and cardiovascular complications.

### Statistical analyses

This was a prospective case series study. With reference to other studies, this study included a small sample of 20 patients. SPSS 23.0 (IBM, Armonk, NY, USA) was used for statistical analysis. Categorical variables are presented as numbers (n), and numerical variables are presented as mean ± SD or median (minimum, maximum; or IQR).

## Results

**Fig. 2:**
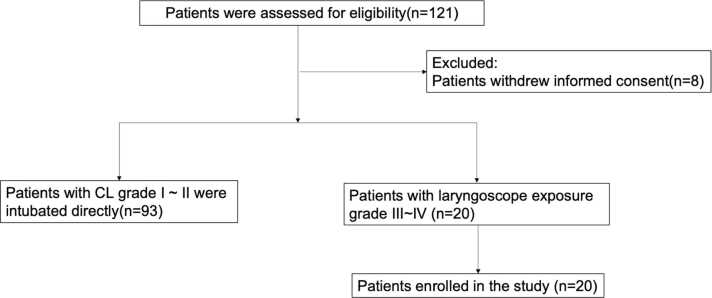
CONSORT flow diagram of patient selection and allocation.

In total, 121 patients with Mallampati classification III–IV were assessed for eligibility, of whom 101 were excluded (Eight due to withdrawal of informed consent and 93 due to a CL grade 1–2, who underwent direct tracheal intubation). Finally, 20 patients with CL grade 3–4 were enrolled (Fig. 2).

### Patient characteristics

The demographic information and baseline characteristics of the patients, such as their ASA classification and Mallampati class, are listed in Table 1. The average age of all patients was 47 ± 11 years, the mean weight was 63 ± 8 kg, and the average BMI was 23.6 ± 2.4 kg·m^−2^. The preoperative status of five patients was classified as ASA class I and that of 14 patients was Mallampati class III. Six patients had a history of hypertension, and one patient had a history of diabetes/anemia/heart disease/undifferentiated connective tissue disease. The average length of stay was 10.6 ± 6.0 days.

**Table 1:** General characteristics of the patients. Values are mean (SD) or number.

Characteristics	n = 20
Age (years), mean (SD)	47 (11)
Sex, (male/female)	7/13
Weight (kg), mean (SD)	63 (8)
BMI (kg·m^−2^), mean (SD)	23.6 (2.4)
ASA grade I/II	5/15
Mallampati class III/IV	14/6
Co-morbidity	
Hypertension	6
Diabetes	1
Anemia	1
Undifferentiated connective tissue disease	1
Heart disease	1
Operation type	
Gynecology	8
Liver	3
Urology	6
Gastrointestinal	2
Pancreatic	1
Length of stay (days), mean (SD)	10.6 (6.0)

### The primary endpoint

**Fig. 3:**
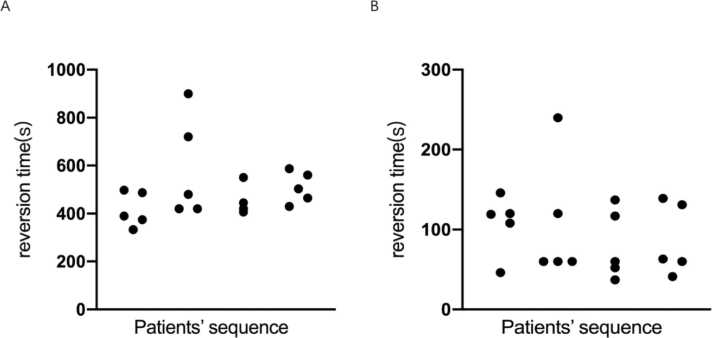
Reversion time(s). (A) The longitudinal axis represents patients’ reversion time from induction. The lateral axis represents all patients’ sequence. (B) The longitudinal axis represents patients’ reversion times from the administration of sugammadex. The lateral axis represents all patients’ sequence.

The primary endpoint was the rate of reversion success within 15 min. All patients (20/20) were reversed successfully within 15 min from the beginning of induction, with 18 patients (18/20) achieving reversion within ten minutes from the beginning of induction. The shortest time to reversion was 333 s, the longest was 900 s, and the median time was 455 s (Fig. 3A). The average awake time of patients was 95.8 s from the time of sugammadex administration, with the shortest time being 37 s and the longest being 240 s (Fig. 3B). The longest time from administration of the muscle relaxant to the restoration of spontaneous breathing was 600 s, and the shortest time was 212 s.

### The secondary endpoint

The Narcotrend reading was 82.52 ± 18.5 after induction and 66.95 ± 13.6 five minutes later. TOF ratio count reached zero within the expected time after the administration of a neuromuscular blocking drug. The median time from anesthesia induction to the restoration of TOF ratio to 90 % was 438 s, and that to the restoration of a Narcotrend reading of 80 was 420 s. The time from induction to tidal volume of above 6 ml·kg^−1^ and the time from induction to recovery of consciousness were the same as the time to the restoration of a Narcotrend reading of 80.

### Safety measurements

**Fig. 4:**
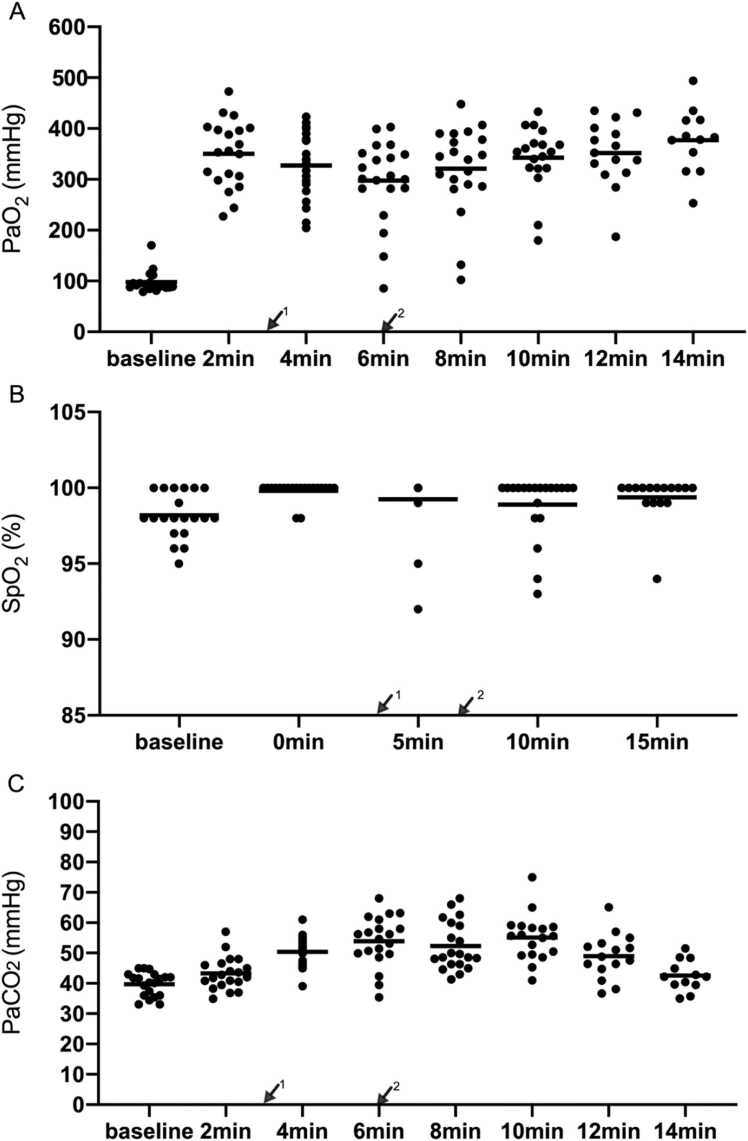
Blood gas results are measured every two minutes from the beginning of induction. Vital signs are measured every five minutes from the beginning of induction. Blood gas results in (A) PaO2, (B) SpO2, and (C) PaCO_2_. ↙^1^: Administration of rocuronium; ↙^2^: Administration of sugammadex.

No hypoxia occurred during reversion. The lowest partial pressure of oxygen (PaO_2_) value from induction to reversion success was 132 mmHg and the lowest PaO_2_ value after reversion was 102 mmHg (Fig. 4A). The average value of baseline SpO_2_ was 98.2 ± 1.5 % and the lowest value of SpO_2_ was 92 % before reversion (Fig. 4B).

**Fig. 5:**
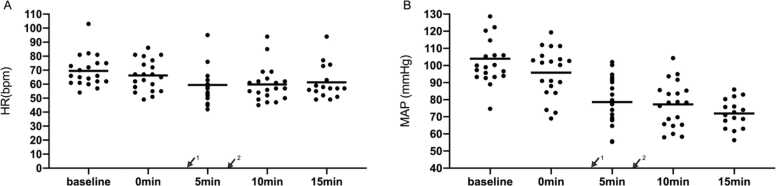
Monitoring results in heart rate (A), mean arterial pressure (B). ↙^1^: Administration of rocuronium; ↙^2^: Administration of sugammadex.

During this period, acceptable hypercapnia occurred. In the context of difficult airway management, permissive hypercapnia is deliberately utilized to mitigate the risk of hypoxemia-related complications while maintaining physiological equilibrium. Elevated PaCO₂ has been demonstrated to induce rapid cerebral vasodilation, thereby increasing cerebral blood flow and intracranial pressure. These hemodynamic changes may precipitate adverse neurological sequelae, including cephalalgia, emesis, and transient visual disturbances. Although early observational studies, such as those by Hickling et al., suggested that extreme hypercapnia (e.g., PaCO₂ levels up to 20 kPa [≈150 mmHg]) could be transiently tolerated in select critically ill populations,^8^ this study established a conservative upper limit of PaCO₂ ≤ 80 mmHg (10.7 kPa) to prioritize patient safety. From induction to reversion success, the highest partial pressure of carbon dioxide (PaCO_2_) value was 68 mmHg and the highest PaCO_2_ value was 65 mmHg after reversion (Fig. 4C). Patients’ cardiovascular vital signs were stable. Changes in mean arterial pressure and heart rate were within the acceptable ranges. The heart rate of three patients was below 50 bpm, which normalized without drug treatment. No patient had a heartbeat above 100 bpm after anesthesia induction. The mean arterial pressure of three patients was below 65 mmHg. The systolic blood pressure of two patients was lower than 90 mmHg, and this was soon relieved after ephedrine treatment (Fig. 5A, B). No side-effects associated with sugammadex occurred, such as unexpected drop in heart rate (during hypercapnia and awakening). All the patients successfully underwent awake tracheal intubation and completed the operation. No other adverse events were observed during the patients’ hospital stay.

## Discussion

In this study, we innovated a new RRI strategy in which HFNO was combined with short-acting anesthetic agents that can provide reversible induction. The present study suggests that the tested RRI strategy can successfully reverse the condition of patients (simulating the CICV scenario) in 15 min without oxygen desaturation when using HFNO.

To our best knowledge, this is the first study to test the feasibility and safety of reversion in CL grade 3–4 patients after induction.

As anticipated, the most critical phase of anesthesia reversal is during intubation, when all administered agents (e.g., propofol, rocuronium) reach their peak plasma concentrations. Our study observed that the time from sugammadex administration to full neuromuscular recovery (TOF ratio ≥ 0.9) ranged from 30 s to 4 min when reversal was initiated at the intubation phase. However, in real-world CICV scenarios, the progressive decline in anesthetic blood concentrations during prolonged intubation attempts implies that the actual reversal time required for full consciousness restoration may be shorter than experimental values.

Optimal reversal timing should precede the onset of irreversible CICV, as neuromuscular blockade reversal itself requires time (e.g., 2–3 min for sugammadex). This proactive approach leverages one key physiological advantages: spontaneous ventilation superiority. Unlike passive ventilation via face mask or laryngeal mask airway (LMA), spontaneous breathing generates negative intrathoracic pressure, enhancing alveolar recruitment and gas exchange efficiency. Therefore, active reversal and awakening protocols should be embedded in CICV management algorithms, even after failed intubation, rather than persisting with futile ventilation attempts.

Many scholars have also pointed out the importance of reversal and studied it from different perspectives. A randomized controlled trial confirmed that rapid sequence induction and intubation using rocuronium followed by reversal using sugammadex enabled earlier reestablishment of spontaneous breathing compared to succinylcholine. The median time from tracheal intubation to spontaneous ventilation was 216 s with rocuronium-sugammadex, and 406 s with succinylcholine. The primary endpoint was the time from tracheal intubation to spontaneous breathing, defined as a respiratory rate of more than 8 bpm and reaching a tidal volume of over 3 ml·kg^−1^ for 30 s.^9^ In our study, spontaneous breathing recovery was defined as reaching a tidal volume of at least 6 ml·kg^−1^, calculated according to standard body weight, which can provide a sufficient gas exchange. The combination of rocuronium and sugammadex seems to have greater advantages, being faster and having fewer contraindications than succinylcholine.

A safe and effective reversal protocol must encompass both the restoration of spontaneous ventilation and the expeditious return of consciousness. Previous studies have demonstrated that patient tolerance of an endotracheal tube (ETT) is a critical factor when resuming spontaneous breathing while maintaining endotracheal intubation, as this can influence tidal volume adequacy and delay the recovery of effective ventilation.

However, Kopman pointed out that in the first few minutes after the induction of anesthesia, the primary factor that determines the possibility of the return of spontaneous respiration may not be the depth of the neuromuscular block, but rather the degree of centrally mediated respiratory depression induced by the opioids, sedatives, and hypnotics used for anesthesia induction.^10^ Naguib used pharmacology simulation models to explore the degree of rapid reversal of neuromuscular blockade using sugammadex, which might improve the recovery of spontaneous ventilation in the CICV setting. The induction drug sequence was fentanyl, followed by propofol, and then rocuronium. The effect of rocuronium was reversed three minutes later using sugammadex. The model simulated preoxygenation for one minute and three minutes, with a fraction of inspired oxygen of 0.6. Some patients did not regain spontaneous ventilation and some experienced hypoxia.^11^ In this study, a CICV model was established. On the premise of the short duration of preoxygenation, fentanyl and propofol were administered in a single injection. Their combined action led to the occurrence of hypoxemia and failure of induced reversal. Unlike the unexpected CICV model, RRI ensures adequate oxygen supply and rapid reversal of drug effects with adequate planning and preparation. HFNO was utilized to deliver efficient preoxygenation and maintain continuous oxygenation throughout the procedure, thereby prolonging the safe apnea duration. TCI of remifentanil and propofol was titrated in real-time based on Narcotrend index and hemodynamic parameters, ensuring precise drug administration. The selected agents—propofol, remifentanil, rocuronium, and sugammadex—are characterized by a rapid onset, short context-sensitive half-times, and predictable pharmacokinetics, which minimize residual effects. Through this multimodal approach—integrating optimized oxygenation, pharmacologic precision, and rapid reversibility—we ensured the safety of RRI implementation in both anticipated and unanticipated difficult airway scenarios. All patients regained spontaneous ventilation and none of them experienced hypoxia.

There are two principles in the implementation of RRI. First and more importantly, it is essential to avoid hypoxemia. HFNO can provide sufficient oxygen and extend the safe apnea time.^6^, ^7^, ^12^, ^13^, ^14^, ^15^, ^16^ Its mechanisms include continuous positive airway pressure in the upper respiratory tract^17^, ^18^ and turbulence caused by the pressure of high-flow oxygen to the glottis.^19^ Our previous study showed that HFNO can maintain oxygenation for 20 min in patients experiencing apnea^7^; therefore, we chose 15 min as the end point of the test to guarantee safety. If the patient is not fully awake within 15 min, the anesthesiologist should conduct mask-assisted ventilation and declare the case a failure.^7^, ^20^ Second, the selected anesthetic agents must be rapidly metabolized or reversed. In the current study, we selected propofol and remifentanil as anesthetic agents because they are fast-metabolizing drugs that enable rapid reversal of anesthesia induction. In our investigation, the TCI effect-site concentration of remifentanil was set to 4 ng·mL^−1^, and that for propofol was 4 μg·mL^−1^, based on a previous study.^21^ Rocuronium 0.6 mg·kg^−1^, a regular induction dose, was chosen in the present study because it can be reversed by sugammadex. This dose is typically lower than the 1.2 mg·kg^−1^ commonly used for rapid sequence induction dose given to full-stomach patients. We chose the maximum dosage of sugammadex, 16 mg·kg^−1^, based on a previous study^22^ to find the shortest time needed for reversion. This dose is reasonable for immediately reversing muscle relaxation after anesthesia induction, as specified in the drug instructions.

All patients awoke within 15 min from the beginning of induction. Patients’ average waking time was 95.8 s from the time of sugammadex administration, with the shortest time being 37 s, and the longest being 240 s. Following administration of the neuromuscular blocking agent (rocuronium), patients were managed without positive-pressure ventilation (either manual or mechanical). The interval from rocuronium administration to reversion ranged from 212 to 600 s, with "ventilation time" defined as the duration of sustained spontaneous breathing. Throughout the procedure, HFNO was maintained at 60 L/min with 100 % FiO_2_, coupled with jaw-thrust maneuvers to ensure airway patency. Its safety has been confirmed in our previous applications (adequate oxygenation and acceptable increase in carbon dioxide).^7^ There were no hypoxia, serious arrhythmias, or hemodynamic disturbances during the reversion.

The RRI strategy demonstrates high safety and is particularly indicated for patients with suspected difficult airways. Importantly, the clinical efficacy of RRI remains robust regardless of the accuracy of preprocedural airway difficulty prediction. Compared to regular induction intubation, RRI offers three distinct advantages: (1) Emergency reversibility: Immediate pharmacologic reversal (e.g., sugammadex for rocuronium) can be initiated if unexpected intubation difficulty arises, preventing progression to CICV scenarios. (2) Adaptable anesthetic depth: RRI maintains sufficient anesthetic depth to facilitate first-attempt intubation success in straightforward airways, while preserving rapid reversibility. (3) Procedural feasibility: This protocol can be reliably implemented by anesthesiologists with standard airway management training, eliminating dependence on highly specialized skills. Our comparative analysis of RRI versus regular induction intubation and awake intubation is presented in Table 2.

**Table 2:** Comparison of regular induction intubation, awake intubation and rapid reversible induction.

	Regular induction intubation	Rapid reversible induction	Awake intubation
**Range of applications**	Non-difficult airway after assessment	Suspected difficult airway	Anticipated difficult airway
**Sufficient depth of anesthesia**	Yes (Four Elements of General Anesthesia)	Yes (Four Elements of General Anesthesia)	Proper sedation before airway establishment
**induction medicine selection**	Regular	Short-acting	Appropriate sedative drugs before airway establishment
**Oxygen supply mode**	Face mask/HFNO	HFNO	Regular-Nasal Cannula/HFNO
**Opioid types**	Many kinds	Remifentanil	Many kinds
**Opioid dose**	High	High	Low
**Sufficient neuromuscular blockade**	Yes	Yes	No
**Controllability**	Poor	Good	Good
**Comfort level**	Good	Good (if not leads to awake intubation)	Poor
**If intubation failure**	Hard to reverse	Easy to reverse	Easy to reverse
**Recovery time**	Long/ fail	Short	Short
**Difficulty of performance**	Easy	Easy	Difficult
**The degree of training**	Basic	Basic	Special
**Special equipment**	No	Target controlled infusion pump	Fiberoptic bronchoscopy

HFNO: High-flow nasal oxygen. N/A: Not applicable.

Existing guidelines for difficult airway management^23^, ^24^, ^25^ predominantly stratify strategies based on preprocedural anticipation of airway difficulty. However, in clinical practice, airway status often remains ambiguous due to dynamic anatomical changes, incomplete preoperative assessments, or emergent conditions, thereby escalating the risk of life-threatening hypoxemia or failed airway rescue. To address this critical gap, we propose RRI strategy tailored for patients with suspected difficult airways. This approach transcends binary "anticipated vs. unanticipated" classifications, offering a unified solution for uncertain or evolving airway challenges.

The ASA guidelines^25^ recommend the use of a face mask, laryngeal mask airway, or tracheal tube in emergencies involving inadequate ventilation or failed intubation. However, we propose modifying this protocol when implementing the RRI strategy: if RRI is initiated during induction, actively awakening the patient should supersede tracheal intubation as the primary rescue measure.

This recommendation aligns with Plan C of the 2015 Difficult Airway Society (DAS) guidelines,^23^ which prioritizes waking the patient upon failed intubation. We further suggest that Plan C be revised to state: "If face mask ventilation is impossible and paralysis has been administered, actively awaken the patient—particularly when RRI is employed—instead of proceeding to invasive airway techniques only after the failure of face mask ventilation."

Rationale for RRI Integration in CICV Scenarios:

• Simultaneous Action: During a CICV crisis, the reversal of anesthesia and preparation for emergency surgical airway access (e.g., cricothyrotomy) should occur concurrently to minimize time-to-rescue.
• Prophylactic RRI Application: For patients with anticipated difficult airways, RRI-compliant anesthetics (e.g., propofol-remifentanil-rocuronium/sugammadex) are administered to ensure rapid reversibility.
• HFNO Optimization: HHFNO maintains adequate oxygenation and extends the safe apnea window, enabling deliberate decision-making during failed intubation sequences.

Escalation Protocol:

• Failed Glottic Exposure: If glottis visualization is inadequate, reversing anesthesia (e.g., sugammadex administration) takes precedence over repeated intubation attempts.
• Failed Plan B (LMA): When both intubation and LMA insertion fail, immediate reversal of neuromuscular blockade and sedation is mandated to avoid further muscle relaxation that could exacerbate ventilation failure.

There are several limitations in the current study. First, the CICV condition was simulated with CL grade 3–4 patients. In clinical practice, in the case of a CL grade 3–4 patient, a video laryngoscope, such as a C-MAC D blade or fiberoptic intubation, can be successfully used. Since CICV cases in clinical practice are very rare, it is difficult to study in short term. Simply antagonizing muscle relaxants without using RRI protocol during induction may not achieve reversal of anesthesia induction. In the present study, the simulated condition of CICV gave us exact data and guaranteed the safety of the patients. Second, only 20 participants were enrolled in the present study. A larger sample size is necessary to explore the feasibility and safety in other patient groups. Patients with ASA>II and severe preexisting comorbidities (cardiovascular, renal, etc.) were excluded from the inclusion criteria. These patients are at a high risk of complications associated with apnea and hypoxemia. Among these patients, the ability to safely awaken and resume autonomous ventilation needs to be further confirmed in subsequent work. Third, HFNO is necessary to implement the RRI strategy, which is absent in many hospitals. Fourth, sugammadex is expensive and would increase the cost of the procedure. However, it is essential for patients in life-threatening conditions due to failed intubation. Moreover, RRI is only a precautionary strategy, and only a few cases of unanticipated difficult airway require sugammadex.

## Conclusions

In conclusion, this study introduces an innovative RRI strategy for managing suspected difficult airways. The average waking time of patients was 95.8 s from the time of sugammadex administration. The rate of reversion success was 100 %. These results position RRI as a prophylactic strategy for high-risk airway management. Crucially, when unanticipated difficult airways develop during induction (prior to or during CICV progression), immediate reversal via RRI should supersede repeated intubation attempts.

## CRediT authorship contribution statement

**Xiaoying Chi:** Writing – review & editing, Writing – original draft, Software, Investigation, Data curation, Conceptualization. **Yichen Fan:** Writing – original draft, Software, Resources, Data curation. **Xiao Zhang:** Resources, Investigation, Formal analysis, Conceptualization. **Yi Qin:** Writing – original draft, Formal analysis, Data curation. **Jie Xiao:** Project administration, Funding acquisition. **Zhenling Huang:** Writing – original draft, Methodology, Investigation, Formal analysis, Conceptualization. **Diansan Su:** Writing – review & editing, Project administration, Methodology, Funding acquisition, Conceptualization. All authors have read and agreed to the published version of the manuscript.

## Consent for publication

Written informed consent has been obtained from the patients to publish this paper.

## Ethical statement

The study was conducted by the Declaration of Helsinki, and approved by the Ethics Committee of Renji Hospital (RenJiH[2018]01).

## Funding

This work was supported by grants from the 10.13039/501100001809National Natural Science Foundation of China (Nos. 81771133, 81970995, U21A20357, 32170995), Shanghai Municipal Science and Technology Commission Funding (21S31900100), Renji Hospital Clinical Innovation Foundation (PYII20-09, RJTJ-JX-002, RJPY-DZX-007), and Shanghai Municipal Education Commission-Gaofeng Clinical Medicine Support (20191903).

## Declaration of competing interest

The authors declare that they have no known competing financial interests or personal relationships that could have appeared to influence the work reported in this paper.

## Data Availability

Datasets used or analyzed during the current study are available from the corresponding author upon reasonable request.
